# Successful combination of endoscopic pyloromyotomy and balloon dilatation for hypertrophic pyloric stenosis in an older child: A novel procedure

**DOI:** 10.1186/s40792-016-0274-y

**Published:** 2016-12-03

**Authors:** Satoshi Yokoyama, Shiro Uyama, Hiroyoshi Iwagami, Yukitaka Yamashita

**Affiliations:** 1 Department of Pediatric surgery, Japanese Red Cross Society, Wakayama Medical Center, 4-20 Komatsubara-dori, Wakayama, 640-8558 Japan; 2 Department of Gastroenterology and Hepatology, Japanese Red Cross Society, Wakayama Medical Center, 4-20 Komatsubara-dori, Wakayama, 640-8558 Japan

**Keywords:** Hypertrophic pyloric stenosis (HPS), Endoscopic pyloromyotomy (EP), Endoscopic pyloric balloon dilation (EPBD)

## Abstract

**Background:**

Hypertrophic pyloric stenosis (HPS) is a rare cause of gastric outlet obstruction beyond infancy. Ramstedt pyloromyotomy remains the gold standard treatment for HPS. This type of HPS can be managed successfully with pyloromyotomy under laparoscopic or open procedures. Endoscopic pyloric balloon dilation (EPBD) has not been recommended in the treatment of HPS, and there are only a small number of reported cases who had had successful endoscopic pyloromyotomy (EP) for HPS only in infants.

**Case presentation:**

The patient was suspected of having HPS when the patient was 1 year old after infancy. However, his parents thought that the vomiting and poor sucking were caused by Down syndrome-associated muscular hypotonia. Since then, no additional tests have been performed at their request. At 6 years of age, he was readmitted to our department because of persistent vomiting and failure to thrive, and HPS was diagnosed again. However, it was unknown whether the HPS had been persisting since infancy or was acquired. The first EPBD was slightly effective but did not remain effective for a long time. When the second EPBD was performed in combination with EP, the amount and frequency of vomiting were reduced dramatically.

**Conclusions:**

The combination of EP and EPBD procedure may represent a safe, effective, and minimally invasive option for selected HPS patients in whom laparotomy would pose a significant risk or who do not respond to conventional medical treatment. To our knowledge, this is the first report to describe combination treatment with EP and EPBD in an older child with HPS.

## Background

Hypertrophic pyloric stenosis (HPS) is a disorder that usually presents with projectile vomiting in infants. The hypertrophy of the pylorus can progress to near-complete obstruction of the gastric outlet [1, 2]. The Ramstedt pyloromyotomy remains the standard operation for HPS, with a laparoscopic approach favored because of the shorter recovery time and lower rate of wound complications [3–5]. Endoscopic pyloric balloon dilation (EPBD) has not been recommended in the treatment of HPS because of reports of its ineffectiveness [6]. However, successful dilation in selected cases of HPS has also been reported [7], as have a number of recent case series of successful endoscopic pyloromyotomy (EP) for HPS in infants have been reported [8, 9].

Here, we report a case of HPS in an older child successfully treated with a combination of EP and EPBD. To our knowledge, this is the first report to describe combination treatment with EP and EPBD in an older child with HPS.

## Case presentation

The patient was diagnosed with Down syndrome after birth. At the age of 4 months, he began to experience frequent non-bilious vomiting along with decreased weight gain. His parents did not want him to undergo any diagnostic tests before he became a year old.

At 1 year of age, he was admitted to our department with long-standing multiple episodes of non-bilious vomiting along with poor weight gain. An upper gastrointestinal (UGI) series showed no evidence of gastroesophageal reflux disease (GERD). However, obstruction at the pylorus, which met the criteria for hypertrophic pyloric stenosis (HPS), was present, and gastric emptying was significantly delayed. Abdominal ultrasonography (US) showed the muscle layer to be 3-mm-thick, and the pylorus to be 15-mm-long. However, his parents thought that the vomiting and poor sucking were caused by Down syndrome-associated muscular hypotonia and did not want the patient to undergo any tests. Therefore, the patient was observed and given only conservative treatment with oral H_2_ blockers and enterokinetic drugs. Non-bilious vomiting along with poor weight gain persisted for 5 years.

**Fig. 1:**
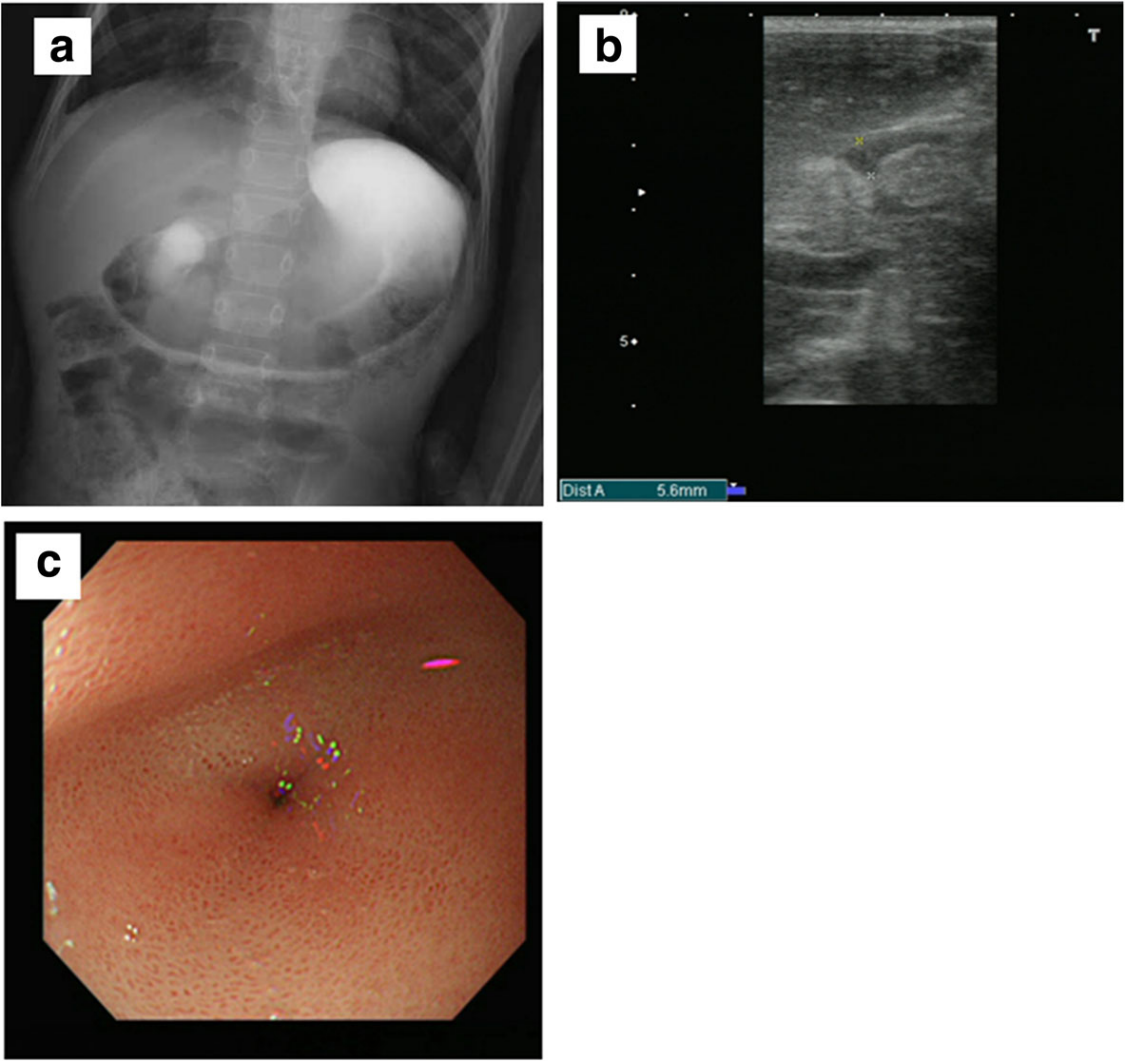
**a** UGI series showed dilated stomach with no contrast seen distally. **b** US showed the muscle layer to be 5.6 mm thick, and the pylorus to be 15 mm long. **c** EGD demonstrated mild erythema of the distal esophagus, markedly enlarged, thickened, and the pin-hole opening pyloric stenosis that did not allow the passage of an endoscope

At 6 years of age, he was readmitted to our department because of persistent vomiting and failure to thrive. Weight was 10.3 kg (−3 SD), height was 94.7 cm (−4.1 SD). His laboratory analyses showed almost normal range. Gastric outlet obstruction (GOO) was observed on an UGI series (Fig. 1a). US showed the muscle layer to be 5.6-mm-thick, and the pylorus to be 15-mm-long (Fig. 1b). An esophagogastroduodenoscopy (EGD) demonstrated mild erythema and marked thickening of the distal esophageal mucosa, consistent with reflux esophagitis, and a pin-hole opening in the pylorus that did not allow the passage of an endoscope (Fig. 1c). Finally, the patient was diagnosed with HPS. However, it was unknown whether the HPS had been persisting since infancy or was acquired.

Ramstedt pyloromyotomy is generally performed in infants and is seldom performed in six-years-old children. Therefore, it was unknown whether or not Ramstedt pyloromyotomy was definitely effective. At first, we attempted EPBD because the patient obviously had Down syndrome-associated muscular hypotonia in addition to chronic respiratory infection. Under general anesthesia, the pediatric endoscope could not be passed through the pylorus. A balloon catheter was introduced transorally into the stomach under fluoroscopic guidance and guided into the pyloric canal. Catheter balloons (Hercules® 3 Stage Wire Guided Balloon, Cook Medical, Winston-Salem NC, USA) of increasing diameters (during the first balloon dilatation, the balloon was gradually inflated to 8 mm, 12 mm, and 15 mm) were inserted through the biopsy channel of a 9.9 mm endoscope (GIF Q260J®, Olympus Optical Co.) and inflated with the use of a pressure gauge system for 60–120 s. The endoscope was able to be easily passed through the pylorus after the third session. After the first balloon dilatation, vomiting gradually decreased in frequency and amount. However, it recurred after a period of 2 months post-dilation. Repeat UGI series showed that the pyloric stenosis was not improved.

**Fig. 2:**
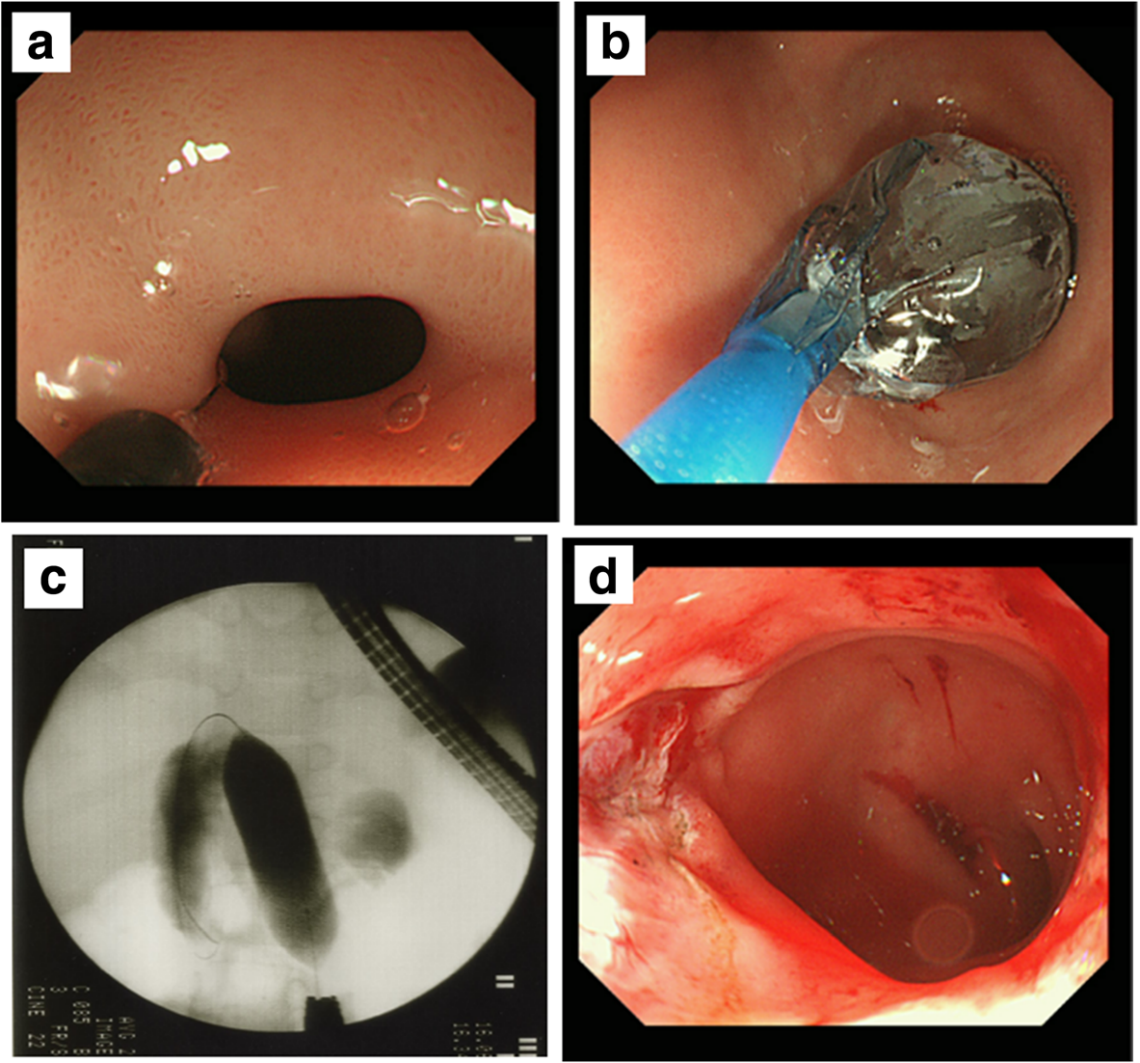
**a** Two incisions were made in the pylorus along the antimesenteric border from the antrum to the duodenal bulb with an electrosurgical needle knife. **b** EPBD was then performed in the same manner and with the same devices as the procedure for initial balloon dilation. **c**, **d** The muscle layer was slowly loosend and split bluntly along the incisions. Compression with the balloon dilator also provided good hemostasis

**Fig. 3:**
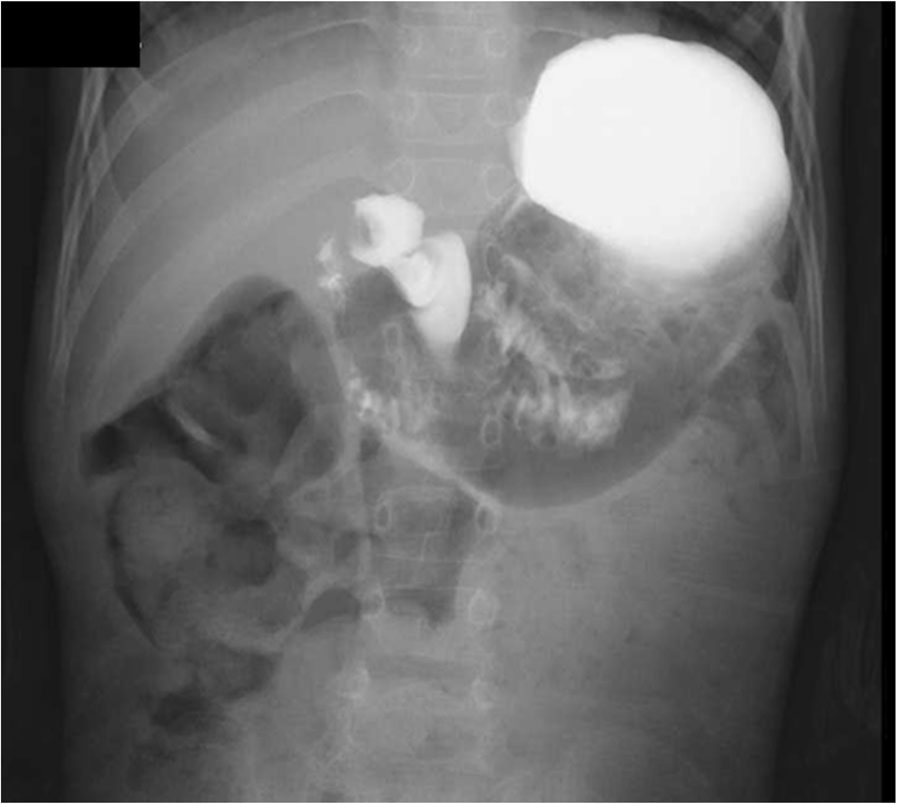
UGI series showed smooth passage of contrast through the pylorus

Six months after the initial dilation, we treated the patient with a combination of EP and EPBD. Under general anesthesia, the same endoscope (GIF Q260J, Olympus Optical Co., Ltd., Tokyo, Japan) was used. The pylorus did not allow passage of the endoscope. Pyloromyotomy was performed with an electrosurgical needle knife (KD-611L IT Knife2®, Olympus Optical Co.) and an electrosurgical generator (VIO® 300 D, Erbe USA, Marietta CA, USA), with settings of cut 30 and blend 3. Two incisions were made in the pylorus along the antimesenteric border from the antrum to the duodenal bulb (Fig. 2a). The incisions were deepened (2 mm) until longitudinal muscle was exposed. EPBD was then performed in the same manner and with the same devices as the procedure for initial balloon dilation (Fig. 2b). The muscle layer was slowly loosened and split bluntly along the incisions. Compression with the balloon dilator provided good hemostasis (Fig. 2c, d). At the end of the procedure, hemostasis was confirmed. Introduction of water-soluble radiographic contrast confirmed free flow across the pylorus. Thereafter, the endoscope was easily passed through the pylorus into the duodenum. Feeding with clear liquids was begun 2 h after the procedure. The patient was discharged the next day. The second EPBD dramatically reduced the amount and frequency of vomiting, and repeat UGI series showed that the pyloric stenosis was improved (Fig. 3). The patient was doing well and had gained weight on follow-up at 6 months.

### Review of the English-language literature (Table 1)

**Table 1:** Overview of reports on non-surgical techniques for HPS

Author, reference	*n*	Age	Type of non-surgical techniques	Complication	Outcome
[6]	6	4–7 weeks	EPBD	None	No successful dilatation
[7]	1	3.5 months	EPBD	Redo needed	Successful
[8]	10	3–7 weeks	EP	None	Successful
[9]	9	4–10 weeks	EP	1 redo needed	Successful
Our case	1	6 years	EP plus EPBD	None	Successful

*EPBD* endoscopic pyloric balloon dilatation

*EP* endoscopic pyloromyotomy

A PubMed search on November 2016 for articles published since 1965 with the key words *hypertrophic pyloric stenosis*, *endoscopic pyloromyotomy*, and *balloon dilatation* yielded only four articles in the English-language literature. These publications were all reviewed. Only two papers describing 19 cases of EP for HPS were found. Ibarguen-Secchia et al. reported ten infants who underwent EP without any adverse events [8]. Zhang et al. reported nine cases in China; one patient underwent a second EP because of recurrent postoperative vomiting [9]. Two papers described seven cases of EPBD for HPS. Hayashi et al. reported six cases; however, EPBD for HPS was not reliable or safe because of the inadequate split and possible full-thickness disruption of the pylorus [6]. Ogawa et al. reported a case of successful EPBD for HPS [7].

## Discussion

The combination of EP and EPBD may represent a safe, effective, and minimally invasive option for HPS. HPS is the most commonly encountered surgical disease in children. Ramstedt pyloromyotomy remains the gold standard treatment for HPS [1–3]. Laparoscopic pyloromyotomy is a minimally invasive version of the Ramstedt procedure that has been associated with a lower incidence of postoperative emesis and a shorter hospital stay, but occasionally results in incomplete pyloromyotomy [4, 5]. Because data on conservative management is very limited, and because surgery is both safe and effective, it is suggested that conservative management should only be indicated in infants in whom a surgical approach is either not advisable or feasible [2].

Endoscopic or image-guided balloon dilation of the pylorus has been also described for patients with HPS. However, preliminary reports documented an unacceptably high rate of both perforation and failure to relieve the obstruction [7], limiting its utilization on wide scale. Like the use of atropine, it may still have a role in children with a significant contraindication to surgical pyloromyotomy.

Recently, however, a few cases series of successful EP have been reported for HPS in infants (Table 1). Although the outcome of balloon dilatation for HPS in infants is not preferable, it has been reported that balloon dilatation is effective for gastric outlet obstruction (GOO) and pyloric stenosis (PS) in adults or older children [10–14]. However, the combination of EP and EPBD for HPS has not been reported on.

In our case, the patient was not an infant but an older child. The patient was suspected of having HPS when the patient was 1 year old after infancy. However, it was unknown whether the HPS was congenital or acquired. Therefore, it was unknown whether or not Ramstedt pyloromyotomy was definitely effective. The first EPBD was slightly effective but did not remain effective for a long time. When the second EPBD was performed in combination with EP, the amount and frequency of vomiting were reduced dramatically. We suggest that the combination of EP and EPBD may be also superior to open and laparoscopic pyloromyotomy for selected HPS. Therefore, we expected that the combined therapy would be effective. It is difficult to conclusively prove that the use of the balloon along with the pylorectomy made any difference.

## Conclusions

To our knowledge, however, no study has reported successful treatment of HPS with combination of EP plus EPBD in an older child. This combination procedure may represent a safe, effective, and minimally invasive option for selected HPS patients in whom laparotomy would pose a significant risk or who do not respond to conventional medical treatment. Given the patient’s good condition and absence of any clinical gastric outlet symptoms, confirmation of the reliability of this combination method for selected HPS cases requires more experience with the procedure and long-term follow-up.

## Abbreviations

EGD: Esophagogastroduodenoscopy
EP: Endoscopic pyloromyotomy
EPBD: Endoscopic pyloric balloon dilation
HPS: Hypertrophic pyloric stenosis
UGI: Upper gastrointestinal
